# Research Progress in Exosome-Based Nanoscale Drug Carriers in Tumor Therapies

**DOI:** 10.3389/fonc.2022.919279

**Published:** 2022-06-21

**Authors:** Wei Fu, Tingting Li, Hongbo Chen, Shu Zhu, Changkai Zhou

**Affiliations:** ^1^ Department of Pharmacy, Tongji Hospital, Tongji Medical College, Huazhong University of Science and Technology, Wuhan, China; ^2^ Department of Plastic and Cosmetic Surgery, Tongji Hospital, Tongji Medical College, Huazhong University of Science and Technology, Wuhan, China; ^3^ Department of Medical Ultrasound, Tongji Hospital, Tongji Medical College, Huazhong University of Science and Technology, Wuhan, China; ^4^ Department of Burn and Plastic Surgery, Affiliated Hospital 2 of Nantong University, Nantong First People’s Hospital, Nantong, China

**Keywords:** exosomes, nanoscale drug carriers, drug-loading strategy, tumor therapy, miRNA

## Abstract

Current antitumor treatment methods have several reported limitations, including multidrug resistance and serious adverse reactions. Targeted drug delivery systems are effective alternatives that can help healthcare providers overcome these limitations. Exosomes can serve as a natural nanoscale drug delivery system, with the advantages of high biocompatibility, low immunogenicity, and efficient tumor targetability. In this paper, we discuss the biological characteristics of exosomes, summarize the drug-carrying mechanisms of exosome-based drug delivery systems, and examine the potential role and applicability of exosomes in clinical tumor treatment approaches. This review can be used as a guideline for the future development of exosome-based delivery systems in clinical precision tumor treatment.

## Introduction

Cancer refers to a range of diseases that are characterized by the unregulated growth of malignant cells that can proliferate beyond the original malignant site. Current cancer treatment options include surgical interventions, immunotherapy, chemotherapy, and radiation therapy or a combination of these options. Existing treatment methods are generally nonselective and can damage healthy normal tissues at the target site, which is associated with unintended severe side effects (1). In fact, adverse effects induced by chemotherapeutic drugs on healthy tissues and organs are partially responsible for the high mortality rate of cancer patients (2). Therefore, it is desirable to develop novel therapeutic strategies for the precise elimination of cancer cells, thereby reducing the potential for adverse side effects while achieving high therapeutic effectiveness.

Exosomes are nanosized vesicle structures that are secreted from cells and are abundant in all fluids and tissues of the human body (3–5). Bioactive substances are transported from donor cells to recipient cells through exosomes, such as nutrients required for cell growth, and exosomal transport plays an important role in the communication between cells (6). Owing to this targeted transport characteristic, exosome-mediated drug delivery can circumvent the P-glycoprotein drug efflux system, which can mitigate the natural drug resistance response in the body (7). Furthermore, exosomes can specifically bind to receptors on the surface of target cells, allowing exosome-mediated drug transport to be specific to a cell or tissue. In addition, the high biocompatibility and low toxicity of naturally derived exosomes make exosome-based delivery systems superior to synthetic drug carriers in clinical applications (8).

This review is intended to provide a reference for the development and application of exosomes as effective nanoscale drug carriers in tumor therapy by detailing the advantages and challenges that have been reported in recent research.

## Characteristics and Biological Functions of Exosomes

### Basic Characteristics of Exosomes

**Figure 1:**
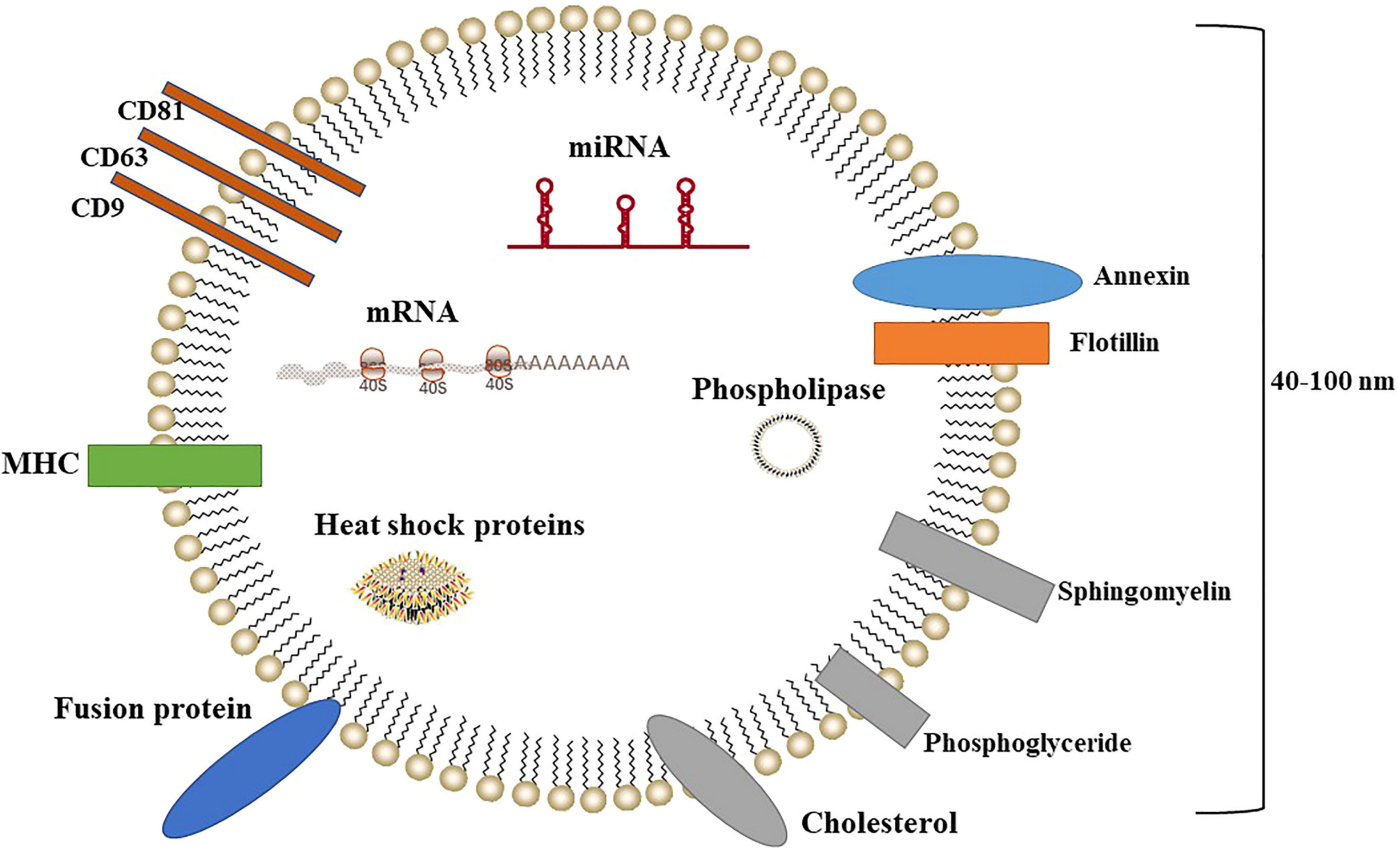
Exosome structure.

Exosomes are intracellular vesicular bodies (40–100 nm in diameter) that are secreted from the cell after the fusion of intracellular multivesicular bodies (MVBs) to the cytoplasmic membrane (**Figure 1**). Exosomes were first discovered in 1983 (9) as small vesicles isolated from sheep erythrocyte supernatant by ultracentrifugation. Exosomes have been subject to increasing scholarly and public attention over the past four decades (10, 11). Exosomes are characterized by lipid bilayers with a rounded or cupped shape. They are widely found in biological fluids such as blood, urine, saliva, and cerebrospinal fluid (12).

Initially, exosomes were considered to function as a “cellular trash bag” that removed unnecessary components from cells. However, later studies reported that exosomes can mediate intercellular communication in many biological processes, and ongoing research has gradually elucidated the important and varied biological functions of exosomes (13, 14). Exosomes are formed by the following processes: the cytoplasmic membrane indents into the cell to form intracellular vesicles, and multiple vesicles fuse with each other to form early endosomes; the early endosomes invaginate again to enclose the intracellular fluid or material and form lumen internal vesicles (i.e., MVBs). Finally, MVBs fuse with the cytoplasmic membrane and release small vesicles into the extracellular space, forming exosomes (15). Exosomes are mainly composed of proteins and lipids, such as fusion proteins, transport proteins, heat shock proteins, CD proteins (i.e., CD9, CD63, and CD81), phospholipases, cholesterol, sphingomyelin, and phosphoglyceride (16). In addition, exosomes contain abundant messenger RNA (mRNA), microRNA (miRNA), small interfering RNA (siRNA), and other genetic materials (17).

Because mRNA can be expressed in target cells, exosome-mediated intercellular communication may be involved in the transfer and expression of genetic information. Exosome-transferred miRNAs have been shown to induce functional changes in target cells. Mittelbrunn et al. (18) found that miRNAs from T-cell exosomes caused the repression of target genes in dendritic cells, and miRNAs in exosomes secreted from Epstein–Barr virus–transfected B cells affected the repression of target genes in dendritic cells.

### Biological Functions of Exosomes

With the rapid development of analytical technologies such as proteomics and gene sequencing, the complex characteristics of exosomes have been subject to continuous investigation and identification. A series of studies has revealed that exosomes are not simply cell debris but subcellular structures secreted by cells that act as specific carriers for the transfer of genetic material and information between cells (19, 20). Exosomes can specifically bind to target cells in a variety of ways. Ligands on the surface of exosomes can bind to target cell receptors, and their membrane or internal proteins can bind to target cell receptors following enzymatic hydrolysis or to target cell receptors *via* fusion with target cells or endocytosis. The material within the exosome can then be transferred to exert biologically active effects. Iaccino et al. (21) studied exosomes obtained from multiple myeloma samples and found that these exosomes expressed immunoglobulin receptors on their surfaces that could bind to a unique binding peptide.

Exosomes are now considered promising drug delivery vehicles due to their small size, high biocompatibility, and reduced toxicity in comparison with synthetic nanoformulations. Additionally, anticancer drugs contained in exosomes have been shown to exhibit improved pharmacokinetic and pharmacodynamic properties and enhanced anticancer activity *in vivo* compared to free-drug administration. Similarly, the loading of therapeutic nucleic acids such as siRNA into exosomes protects the RNA from nucleases and increases cellular uptake, thus improving the therapeutic effect compared to free RNAs that are rapidly degraded by nucleases. Notably, exosomes can cross the blood–brain barrier and penetrate deep tissues with improved efficacy compared to synthetic nanocarriers. Thus, exosomes are an effective and necessary tool for delivering anticancer therapeutics.

## Characteristics of Exosomes as Nanocarriers

### Methods for Loading Exosomes

Mounting evidence has demonstrated that exosomes are an excellent candidate platform for cargo delivery in clinical applications. To date, various strategies have been developed for loading materials into exosomes, such as incubation, sonication, electroporation, and chemical conjugation (22–24).

#### Incubation

The simplest cargo-loading method is incubation, by which the desired cargo is allowed to diffuse into an exosome or exosome-secreting cell *via* a concentration gradient (25). Cargo diffuses across cell and exosomal membranes to then be packaged within exosomes. Two primary incubation methods have been developed: direct incubation and exosome donor cell-mediated incubation followed by isolation of drug-carrying exosomes. The former is suitable for drugs that can interact with the lipid layer of exosomal membranes, while the latter is suitable for small-molecule chemical drugs with low cytotoxicity. Didiot et al. (26) coincubated U-87 glioblastoma with hydrophobically treated small kinetic RNA. The hydrophobic end of siRNA binds to cholesterol on the membrane of U87 glioblastoma-derived exosomes, mediating its entry into exosomes. This coincubation method can yield 10%–50% of siRNA-loaded exosomes. Incubation of exosome donor cells with drugs is another way. Donor cells are treated with the drug, leading to the secretion of loaded exosomes. A combination of paclitaxel and doxorubicin was loaded into exosomes from ovarian cancer cells and breast-to-lung metastatic cells, enabling the inhibitory effects on tumor growth (27).

Incubation loading methods have emerged to load various cargos into exosomes. The main advantages of this strategy are that it is simple and causes only minor damage to exosome integrity. Some disadvantages are that the loading efficiency is limited and the loading quantitation is difficult to control due to physicochemical properties of potential cargo and created exosomes.

#### Electroporation

Electroporation is a strategy for loading cargo into exosomes with assistance from electrical fields that produce micropores on the exosomal membrane to enhance permeability. Although drugs can diffuse into exosomes *via* incubation, drug loading efficiency can be significantly enhanced using electroporation.

Because high-voltage pulses can penetrate the exosomal membrane, subjecting the mixture of drug and exosomes to a certain voltage can induce drug loading. For example, doxycycline-loaded exosomes generated by electroporation have been reported to target tumor tissues and inhibit tumor growth significantly without exerting observable toxicity to other tissues (28). Kim et al. (29) introduced antisense miRNA oligonucleotides targeting miR-21 into exosomes by electroporation to treat glioblastoma. Electroporation has also been utilized to directly load nucleic acids and nanomaterials into exosomes. Izco et al. (30) loaded short hairpin RNA (shRNA) into murine dendritic cell-derived exosomes, which decreased α-synuclein aggregation and alleviated dopaminergic neuron loss in Parkinson's disease (PD) models.

#### Transfection

Transfection is the most common strategy for stably loading nucleic acids, proteins, and peptides into exosomes. In this approach, transfection reagents are used to load drugs into exosomes. Cell transfection can be achieved by using the calcium phosphate method or by a commercially available lipid transfection reagent such as Lipofectamine. Choi et al. (31) found that exosomes from osteogenic precursor cells were enriched in let-7 miRNAs under physiological conditions. The introduction of miRNA inhibitors into exosomes by transfection can modulate bone differentiation and improve motor nerve-related diseases and tumor treatment outcomes. Furthermore, Kojima et al. (32) transduced human embryonic kidney cell line 293 (HEK293) cells with designed plasmids to generate catalase mRNA-loaded exosomes that targeted central nervous system (CNS) cells to treat Parkinson’s disease. In addition, exosomes can also be directly transfected with nucleic acids by chemical treatment. Pi et al. (33) isolated exosomes from HEK293 cells and transfected exosomes with siRNA using a heat shock protocol.

The ectopic expression of desired materials or molecules that facilitate specific cargo sorting *via* transfection has emerged as a common strategy. However, the loading efficiency achieved using this method remains relatively low as the cargo-sorting mechanisms are difficult to manipulate intracellularly and direct chemical transfection always induces impurities.

#### Ultrasound

Ultrasound is a physical strategy by which an extra mechanical shear force is applied to weaken the exosomal membrane, which promotes loading of cargo such as drugs, proteins, and nanomaterials. The mechanical shear force generated during sonication disrupts the integrity of the exosomal membrane. The drug enters the exosome during membrane deformation, and the subsequent incubation process restores the integrity of the cell membrane.

Li et al. (34) found that after mixing the drug and exosomes, the drug could be effectively loaded into the exosomes following ultrasound, and the structure and composition of the exosomal membrane did not change significantly after ultrasound. Similarly, Salarpour et al. (35) used sonication and incubation to load paclitaxel (PTX) into U-87 cell-derived exosomes to treat glioblastoma multiforme. Experimental data demonstrate that PTX-loaded exosomes (exoPTX) were more toxic to glioblastoma multiforme cells than free PTX solution.

Recent reports have shown that ultrasound is an effective method for loading drugs into exosomes. However, sonication-induced membrane damage remains a primary roadblock for scaling up this method.

#### Extrusion

Extrusion is a physical process by which an exosome and cargo mixture are subjected to pressure in an extruder mechanism to induce membrane recombination. The exosome and drug mixture are loaded into a liposome extruder with multiple pore sizes. The pore size of the extruder pore membrane is 100–400 nm, which allows the drug to enter the exosomes *via* extrusion. Kalimuthu et al. (36) mixed PTX with bone marrow mesenchymal stem cells and achieved drug loading using the extrusion method. The drug-loaded exosomes obtained by this method not only efficiently target cancer cells but also exhibit reduced cardiotoxicity and neurotoxicity associated with chemotherapeutic drugs compared to free-drug administration (36). Extrusion has high loading capacities, but a rearrangement of exosome surface structure possibly changes their integrity.

To date, a growing number of approaches have been developed and applied to induce exosomal cargo loading. It remains difficult to efficiently load desired materials into exosomes while minimizing exosomal surface damage. **Table 1** compares the advantages and disadvantages of commonly used drug-loading methods. Although various studies have reported successful drug loading using various methods, the appropriate selection of a candidate method depends on the properties of the target drug and the source and properties of the exosomes (37).

**Table 1:** Primary strategies for loading cargos into exosomes.

Types of strategies	Types of cargos	Advantages	Disadvantages
Incubation	Drugs, nucleic acids, proteins, peptides, nanomaterial	Easy operation; Does not require special equipment; Minimal destruction to carrier exosome	Low loading efficiency; Difficult to control;The amount of drug that diffuses into the cells and loads into the exosome is unpredictable
Electroporation	Drugs, nucleic acids, proteins, peptides, nanomaterials	High loading efficiency; Loading large biomolecules	Influence exosome integrity; Cargo aggregation
Transfection	Nucleic acids, proteins, peptides	High loading efficiency for nucleic acids, proteins, and peptides; Stability	Time- and financial consuming; Hard to quantitate
Ultrasound	Drugs, proteins, peptides, nanomaterials	High loading efficiency; Effective method of loading different types of cargo	Disruption of exosome integrity; Attachment of drugs to the surface of exosome
Extrusion	Drugs, nucleic acids, proteins, peptides, nanomaterial	High loading efficiency	Disruption of exosome integrity

## Strategies for Surface Engineering of Exosomes

Despite being naturally derived, exosomes can be modified by various genetic engineering methods. Genetic engineering approaches fuse genes for signaling proteins or polypeptides with those for selected exosomal membrane proteins, thereby conferring cell-targeted specificity that improves the suitability of the produced exosomes for transport, increasing the concentration and duration of effect of the loaded drugs at the tumor site to achieve desirable treatment outcomes.

Several strategies for modification of the exosome have been tested. For example, exosomes obtained from HEK293 were genetically engineered to express GE11 short peptide and miRNA Let-7a (38). The GE11 peptide binds to the epidermal growth factor receptor, which is highly expressed in many epithelial tumors. The engineered exosomes were labeled with 1,1′-dioctadecyltetramethylindole tricarbocyanine iodine and injected intravenously into tumor-bearing mice. The monitoring results of *in vitro* and *in vivo* imaging showed that three times as many GE11 exosomes reached the tumor site than did those of the control group, and the loaded miRNA Let-7a effectively inhibited tumor growth. Similarly, incorporating ligand modifications on the surface of exosomes is another common strategy widely endorsed. For instance, exosomes obtained from HEK293T cells were anchored with lipophilic hyaluronic acid (lipHA-hEVs) as a ligand to target CD44-overexpressing breast cancer cells to deliver doxorubicin. These lipHA-hEVs were shown to enrich doxorubicin specifically in breast cancer cells, thereby reducing the tumor mass by 89% and increasing the animal survival by 50% (39). In addition, receptor protein engineering is another strategy for preparing exosomes for targeted delivery. Recently, a method was described in HEK293 cells that enabled partitioning of reporter proteins into the inner or outer surface of exosome by utilizing specific locations on the tetraspanin proteins for fusion protein engineering. Such methods immensely allow engineering of exosomes to achieve significantly improved targeting capacity.

In summary, all of these methods significantly improve the tumor-targeted drug delivery of the native exosomes and help reduce the toxicity. Therefore, the latest studies focusing on the development of combined targeting strategies with enhanced exosome homing capacity and drug delivery efficiency have reported promising results that provide a foundation for the clinical application of exosome-based drug delivery systems.

## Application of Exosomes as Nanodrug Carriers in Tumor Therapy

### Protein Drugs

Recently, the development of exosome-based vaccines has become a topic of significant interest in clinical research (40, 41). Rivoltini et al. (42) reported that exosomes loaded with tumor necrosis factor α (TNFα)-related apoptosis-inducing ligands promoted cancer cell apoptosis and mitigated tumor development. Similarly, interleukin (IL)-18-rich exosomes promoted proinflammatory cytokine release and monocyte proliferation, inducing specific antitumor immune responses (43). Moreover, Yang et al. (44) found that IL-2-rich exosomes could induce antigen-specific T helper cells I (Th1) polarized and killer T lymphocyte-mediated immune responses, leading to significantly inhibited tumor growth in mice.

Exosomes can also be loaded with protein antagonists. For instance, signal regulatory protein α (SIRPα) inhibits macrophage phagocytosis of tumor cells by interacting with CD47. In tumor-bearing mice, exosomes containing SIRPα antagonists were observed to significantly promote tumor phagocytosis (45).

### Chemotherapy Drugs

The indiscriminate nature of antitumor chemotherapeutic drugs, leading to cell death in both tumor and normal cells, is associated with severe adverse reactions in the body. Therefore, it is necessary to develop alternative effective drug carriers that specifically target tumor cells.

Tang et al. (46) reported that tumor cell-derived microparticles could be used as carriers for chemotherapeutic drugs, and the chemotherapeutic drugs encapsulated in the microparticles exerted strong antitumor effects *in vitro* and *in vivo*. Kim et al. (47) developed an exosome-based formulation of PTX, which is a commonly used chemotherapy drug. They incorporated PTX into exosomes using three methods: incubation at room temperature, electroporation, and mild sonication. The mild sonication method produced the highest loading efficiency and sustained drug release. The cytotoxicity of exoPTX against drug-resistant MDCKMDR1 (Pgp+) cells was increased by more than 50-fold. In addition, exoPTX was found to effectively target cancer cells following injection into mice with lung cancer, demonstrating a strong antitumor effect. Similarly, Saari et al. (48) found that the use of exosomes from cancer cells as drug carriers enhanced the cytotoxicity of transported PTX to cognate prostate cancer cells. Furthermore, modifying exosome surface proteins is a common method for improving the targeting of chemotherapeutic drugs to cancer cells. Tian et al. (49) obtained mature dendritic cells (IMDCs) expressing Lamp2b iRGD peptide (specific for av integrin) using a genetic engineering method, isolated exosomes from IMDCs, and transported doxorubicin to target cells *via* exosome loading. Their results showed that iRGD-containing exosomes effectively targeted av integrin-expressing breast cancer cells and inhibited tumor growth.

### Nucleic Acid Drugs

#### miRNA

miRNAs are noncoding RNAs of approximately 20 nucleotides in length. Posttranscriptional gene silencing is mediated by binding to the target site in the 3′ untranslated region or open reading frame of the target mRNA (50). Since exosomes naturally carry miRNAs, it can serve as a therapeutic application for tumors. For instance, miR-1290 and miR-375 in exosomes were positively associated with overall survival in castration-resistant prostate cancer patients (51). Katakowski et al. (52) and Zhang et al. (53) reported that anti-glioma miRNA (miRNA-146b)-enriched exosomes inhibited glioma growth *in vitro*, while miR-101-enriched exosomes demonstrated the ability to inhibit the invasion and migration of osteosarcoma cells.

The abovementioned studies provide an illustration as to how the combined effects of miRNAs and exosome-based therapies may be valuable, especially for the future treatment of cancer. The instability and degradability of miRNAs hinder their application in tumor therapy. Exosomes are stable vesicles that can carry functional biologically active molecules with high targeting specificity. Therefore, the miRNA-loaded exosome system is expected to play a key role in emerging tumor therapies.

#### siRNA

siRNA is a double-stranded RNA with a length of 21–23 nucleotide pairs. In the cytoplasm, RNA-induced silencing complexes interact with complementary mRNAs, leading to their degradation and gene silencing (54). siRNA is the first choice for treating diseases caused by gene overexpression. However, the negative charge of siRNA limits their ability to cross cell membranes, and siRNAs are readily degradable with short half-lives. Therefore, identifying an efficient and reliable method for delivering siRNAs to target cells will significantly improve the efficacy of siRNA-mediated therapies (55).

Alvarez-Erviti et al. (56) exploited the RNA transport ability of exosomes to successfully conduct gene therapy studies in which exogenous siRNA was delivered in a targeted manner for the first time. They transfected IMDCs with a plasmid encoding the exosomal protein Lamp2b and fused it to the brain-specific peptide rabies virus glycoprotein (RVG). RVG-targeted exosomes were purified from dendritic cell cultures, and Glyceraldehyde-3-phosphate dehydrogenase siRNA was loaded into exosomes by electroporation. Mice were then injected with RVG and Glyceraldehyde-3-phosphate dehydrogenase siRNA-loaded exosomes *via* the tail vein. The results show that this mode of administration can specifically reduce Glyceraldehyde-3-phosphate dehydrogenase expression in the cerebral cortex, striatum, and midbrain of mice. RVG BACE1-siRNA-loaded exosomes reduced *BACE1* gene expression in the cerebral cortex of treated mice by 60% when RVG-loaded exosomes were used to deliver BACE1-siRNA targeting β-secretase. Production of the toxic amyloid Aβ_1-42_ was also significantly reduced. Notably, these exosomes were used as efficient and specific delivery vehicles for siRNA without associated toxicity or immunogenicity observed even with repeated administration, which is an important feature of siRNA clinical applications (57).

#### Other Drug Applications

In addition to the abovementioned biologically active molecules, exosomes can silence the expression of related pathogenic genes by transporting specific gene-editing complexes. Lin et al. (58) constructed exosome–liposome composite nanoparticles to effectively transport the gene-editing complex Cas9/Cas9 to the mesenchymal region and subsequently deliver the Cas9-Cas9 complex by being phagocytosed by mesenchymal stem cells. Emerging findings have demonstrated that composite nanoparticles constructed by genetic engineering have a broad application value in novel tumor therapies.

## The Challenges of Using Exosomes in Therapeutic Drug Delivery Systems

Several clinical trials have been attempted over the past decade using exosome-based drug delivery for cancer therapy. However, several questions on the optimization of exosomes for clinical usage remain unsettled (**Table 2**).

**Table 2:** Advantages and challenges of using exosomes in therapeutic drug delivery systems.

Advantages	Challenges
Nanoscopic size	A large-scale purification method for clinical use is still missing
Low immunogenicity	Efficient cargo loading methods without damaging EV integrity are lacking
High biocompatibility	The exact mechanism of interaction between exosomes and targeted cells needs elucidation
Encapsulation of various cargos	*In vivo* data yet less studied
The ability to overcome biological barriers	Clinical translation for therapeutic delivery is lacking

### Sources

The primary consideration in developing therapeutic exosomes is the cell source. Mesenchymal stem cell-derived exosomes are considered promising for cancer therapy, as they are known to be safe and versatile. To optimize their clinical use, critical technological considerations should be addressed and possible side effects identified.

Some research groups have begun isolating exosomes from several types of fruit or vegetables, including ginger, grapes, and lemons, to address problems related to a mammalian cell-derived exosome (59, 60). These exosomes can load small molecules and mediate therapeutic effects on animal models (61). A phase I clinical trial using curcumin-loaded plant-based exosomes for the treatment of colon cancer was conducted.

### Loading

The natural biogenesis of exosomes can be used to load them with therapeutics endogenously by direct loading in the producer cell, such as incubation, electroporation, transfection, ultrasound, and extrusion. However, a large load may affect exosome stability or lead to exosome aggregation, and the loading efficiency seems to be quite variable. For instance, electroporation may affect the integrity of exosomes or drugs, especially when loading genetic drugs, thus raising the concern of safety risks (62).

Alternatively, cellular nanoporation for delivering the phosphatase and tensin homolog (PTEN) mRNA in a mouse brain tumor model showed improvement in electroporation-mediated loading. Yang et al. (63) transfected cells with several nucleic acids by using focal and transient electrical stimuli to generate nanopores in targeted cells, which efficiently produced exosomes containing the targeted nucleic acids. The systemically administered PTEN-deficient glioma mouse models with PTEN-loaded exosomes showed a promising effect *in vivo* (63).

### Production

The yield of exosomes collected by existing separation technologies cannot meet the standards for clinical use, and the cost of large-scale production is difficult to estimate (64). Serial centrifugation with ultracentrifugation is currently the standard method for exosome production. This method is limited by the risk of macromolecule contaminants, exosome aggregation, time-consuming, and disruption of integrity. To purify exosomes further by ultracentrifugation, a density gradient separation using iodixanol and a sucrose cushion ultracentrifugation step can be applied, leading to higher purity with a minimal risk of sample loss. Furthermore, size-based methods such as tangential flow filtration and size exclusion chromatography are being widely used for exosome isolation and show better yields, integrity, purity, and functionality of exosomes than by ultracentrifugation-based isolation methods.

Finally, methods for transporting and storing drug-encapsulated exosomes are also urgent concerns that require further research. Studies have found that the optimal temperature for storing exosome samples is -80°C, whereas another approach is storing lyophilized powder at room temperature (65, 66).

## Conclusion

Exosomes have great potential for application in targeted tumor therapy due to their unique biological properties. As nanoscale vesicles secreted by cells, exosomes exhibit unique advantages that make them suitable drug carriers. First, exosomes commonly show high stability, high biocompatibility, and low immunogenicity than other drug delivery platforms (67, 68). Second, nanosized exosomes that bear specific surface proteins can penetrate efficiently the target tumor tissue. These features suggest that exosomes can serve as efficient nanocarriers for the delivery of proteins drugs, chemotherapy drugs, and nucleic acid drugs.

However, the exosome-based drug delivery systems have several limitations in sources, loading, and production. For exosomes to be clinically applicable, the production of functionalized exosomes requires cell culture on a large scale with quality control. Furthermore, given the complexity and heterogeneity of tumors, exosome-based tumor therapy approaches are expected to be the most effective when combined with other nanotherapies (69, 70). There is an urgent need to conduct a large number of clinical trials of exosome-based tumor therapies to determine their efficacy and safety. As these technologies progress and we continue to develop a more comprehensive understanding of exosomal characteristics under various conditions, clinical research on and the applicability of exosomes as a nanoscale drug delivery system in tumor therapies will continue to improve.

## Author Contributions

All authors contributed to the design of the study and writing of the article. WF, TL, and HC wrote the main article text and prepared figures. CZ and SZ revised the article critically for important intellectual content and final approval of the version to be submitted. All authors reviewed the article.

## Funding

This work was supported by the Natural Science Foundation of Hubei Province of China (2021CFB348).

## Conflict of Interest

The authors declare that the research was conducted in the absence of any commercial or financial relationships that could be construed as a potential conflict of interest.

## Publisher’s Note

All claims expressed in this article are solely those of the authors and do not necessarily represent those of their affiliated organizations, or those of the publisher, the editors and the reviewers. Any product that may be evaluated in this article, or claim that may be made by its manufacturer, is not guaranteed or endorsed by the publisher.
